# Biosynthetic Gene Clusters from Swine Gut Microbiome

**DOI:** 10.3390/microorganisms11020434

**Published:** 2023-02-08

**Authors:** Leli Wang, Yiru Zhang, Juan Xu, Chuni Wang, Lanmei Yin, Qiang Tu, Huansheng Yang, Jia Yin

**Affiliations:** 1 Hunan Provincial Key Laboratory of Animal Intestinal Function and Regulation, College of Life Sciences, Hunan Normal University, Changsha 410081, China; 2 Institute of Subtropical Agriculture, Chinese Academy of Sciences, Changsha 410081, China; 3 Tianjin Institute of Industrial Biotechnology, Chinese Academy of Sciences, National Center of Technology Innovation for Synthetic Biology, Tianjin 300308, China; 4 CAS Key Laboratory of Quantitative Engineering Biology, Shenzhen Institute of Advanced Technology, Chinese Academy of Sciences, Shenzhen 518055, China

**Keywords:** biosynthetic gene cluster, gut microbiota, pig, arylpolyene, resorcinol

## Abstract

The abuse of antibiotics has become a serious health challenge in the veterinary field. It creates environmental selection pressure on bacteria and facilitates the rapid spread of antibiotic resistance genes. The speed of discovery and application of cost-effective alternatives to antibiotics is slow in pig production. Natural products from biosynthetic gene clusters (BGCs) represent promising therapeutic agents for animal and human health and have attracted extraordinary passion from researchers due to their ability to participate in biofilm inhibition, stress resistance, and the killing of competitors. In this study, we detected the presence of diverse secondary metabolite genes in porcine intestines through sequence alignment in the antiSMASH database. After comparing variations in microbial BGCs’ composition between the ileum and the colon, it was found that the abundance of the resorcinol gene cluster was elevated in the ileal microbiome, whereas the gene cluster of arylpolyene was enriched in the colonic microbiome. The investigation of BGCs’ diversity and composition differences between the ileal and colonic microbiomes provided novel insights into further utilizing BGCs in livestock. The importance of BGCs in gut microbiota deserves more attention for promoting healthy swine production.

## 1. Introduction

Nowadays, the consumption of antibiotics in livestock accounts for 73% of global use [1]. Such high antibiotic use influences the composition of the gastrointestinal microorganisms and imposes a strong selective pressure on bacteria in animals [2].

Antibiotics are used to prevent diseases in swine and are delivered to piglets by two typical approaches in some developing countries [3]. One is adding antibiotics to feed in advance by factories as feed additives. The other is farmers directly mixing antibiotics in watering or feed systems and through intramuscular injection [4]. As a result of recurrent antibiotic exposure, multiple antibiotic resistance genes rapidly spread, which renders hosts susceptible to pathogen infections that are difficult to treat [5,6]. By 2050, about 10 million people around the world could die each year due to antimicrobial resistance [7]. Although many countries have taken positive measures, antibiotic overuse remains an issue of serious concern [8]. In swine production, the withdrawal of antibiotics from feed is practically associated with frequent enteric infection and compromised growth performance [9]. The speed of the discovery of cost-effective alternatives to antibiotics applied in swine production has been slow, partly owing to their variable outcomes in animal trials and unclear modes of action [10].

Highly diverse and complex microbiota exist in the gastrointestinal tract of pigs with distinct longitudinal, radial, and timely distributions [11]. Increasingly, studies have demonstrated that both swine genetics and gut microbial composition play crucial roles in nutrient metabolism and disease resistance [12,13,14]. The pig is one of the most omnivorous of domesticated farm animal species, which easily adapts to various plant-based feed and carcasses [15]. Similar to the human gut, the pig gut is functionally and anatomically diverse in different compartments, which are all colonized with microorganisms [16]. Some sequencing results showed that the microbial composition in the small intestine was quite different from the microbial composition in the large intestine [16]. Specifically, *Escherichia-Shigella*, *Terrisporobacter*, and *Clostridium sensustricto 1* genera were abundant in the ileum, while *Streptococcus*, *Lactobacillus*, and *Clostridium* genera were enriched in the colon [17]. In addition, the community density and richness of the ileum microbiome were much lower than that of the colon microbiome [17]. This was partly due to the physical environments and physiological functions that the microbiome composition varied in different intestinal segments.

Over a long period of evolution, we had questions regarding how intestinal microorganisms cope with the selective pressure of antibiotics in pigs and whether these microbes also produce bactericidal substances that help them survive and reproduce. Could we exploit potential antibiotic alternatives from the highly complex and diverse microbiome in the pig gut to address the agricultural antibiotic crisis? Our hypothesis was that the gut microorganisms could secrete bioactive substances in farm pigs that received high doses of antibiotics. Herein, we focused on microbial natural products in the swine intestine. The investigation of BGCs’ diversity and the differences in composition between the ileal and colonic microbiomes provided novel insights into further utilizing BGCs in livestock. 

## 2. Materials and Methods

### 2.1. Animal Trial and Sample Collection

The piglet trial procedures performed in this study were approved by the Animal Care and Use Committee of the Hunan Normal University. Twenty male and female crossbred piglets (Duroc × Landrace × Yorkshire, 10 males and 10 females) were selected from two litters and raised by a keeper at the Xinwufeng company in Changsha City. All piglets received the classical swine fever virus vaccine and were housed in individual cages. The pig facility was in a standard room with concrete slatted block flooring and underground drainage. The room temperature was maintained at around 28 °C by air conditioners and lamps. After weaning at 21 days, a corn–soybean feed, without antibiotics, was formulated and provided to these piglets according to NRC (2012) requirements (Table 1). All piglets had free access to feed and water during the trial. No illness, diarrhea, or other abnormal behaviors were observed. Twenty piglets were fasted overnight, euthanized through intravenous injection, and then sacrificed at 35 days. The ileum was sampled from a 10 cm section proximal to the ileocecal junction. A 10 cm segment of the colon was cut. The intestinal digesta samples from the ileum and colon were collected in sterilized tubes. Each sample had two replicates. In order to protect the sample quality, the intestinal digesta samples were quickly frozen with liquid nitrogen and kept at −80 °C until metagenomic sequencing.

### 2.2. Metagenomic Sequencing and Bioinformatic Alignment

For metagenomic sequencing, we followed a previously reported method [18]. The bacterial DNA was firstly extracted from the intestinal digesta samples of the twenty piglets based on the manufacturer protocol, using a fecal QiaAmp DNA Stool Mini Kit (Qiagen, Hilden, Germany) [19]. DNA was sheared into fragments of approximately 350 bp and purified from agarose gel. The metagenomic DNA sequencing was performed with a Illumina HiSeq X Ten platform at Shanghai OE Biotech Co., Ltd. (Shanghai, China). Reads were removed from adapters with KneadData tools (v0.5.1) [20], followed by quality-trim and filter. For sequencing data, the NGSQC toolkit (v2.3.2) [21] was used to filter low quality reads, which contained less than 70% bases with Q20, which were trimmed from the 3′ end. Reads with ambiguous bases were also removed. In addition, reads shorter than 70 bp were removed. After cleaning, the average total bases of the ileal samples were 12.90 G with 92,575,530 average reads, while the average total bases of colonic samples were 12.61 G with 84,206,997 average reads. Regarding the sequencing quality, in the ileum, the average of the left base sequence quality was 39.13 and the average of the right base sequence quality was 36.38. In the colon, the average of the left base sequence quality was 39.20, and the average of the right base sequence quality was 35.78. Next, high-quality reads were assembled into contigs using SOAPdenovo software (version 2.0.4, Beijing Genomics Institute Company, Shenzhen, China, http://soap.genomics.org.cn/soapdenovo.html, accessed on 8 September 2019) [22]. The genes of host origin were removed with BWA (v0.7.9) [23]. The CD-HIT (version 4.5.8, http://www.bioinformatics.org/cd-hit, accessed on 9 October 2019) [24] and NR database (version 2018-01-02, https://www.ncbi.nlm.nih.gov, accessed on 9 October 2019) [25] were used for nonredundant gene set building and taxonomy assignment. The biosynthetic gene cluster sequences of secondary metabolites were predicted and annotated against the antiSMASH database [26]. The functional gene abundance in each sample was evaluated. The relationship between a predicted secondary metabolite and a bacterium in the genus level was calculated using Spearman’s rank correlation coefficient and visualized through R package psych v1.8.12 and Cytoscape [27,28]. Lines were used to describe significant correlations (Spearman’s correlation, *p* < 0.05 and absolute coefficient >0.5). The raw data were deposited in the Genome Warehouse in the National Genomics Data Center [10,11], Beijing Institute of Genomics Chinese Academy of Sciences, under accession numbers GWHBOXZ00000000~GWHBOZH00000000, which are publicly accessible at https://ngdc.cncb.ac.cn/gwh, accessed on 26 October 2022.

### 2.3. Statistical Analysis

GraphPad Prism software (version 8.3; San Diego, CA, USA) was used for data analysis and graph visualization. The significant differences between the ileum and colon were analyzed by the two-tailed unpaired *t*-test. Statistical significance was obtained when the probability value was less than 0.05 (* *p* < 0.05, ** *p* < 0.01). No significance is indicated by *n.s.* The data are displayed as means ± *s.e.m.*

## 3. Results

**Figure 1:**
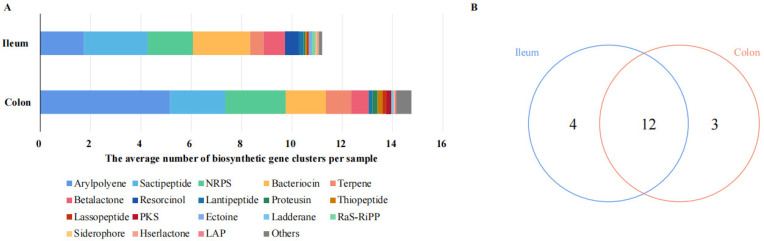
The distribution of BGCs in the porcine intestine. (**A**) The average number of BGCs per sample in the ileal and colonic microbiomes; (**B**) Venn diagram of the specific and shared BGCs in the ileal and colonic microbiomes.

Through the prediction of the pig metagenomics data in the antiSMASH database [29], we detected that there were diverse secondary metabolite genes in the porcine intestine, such as arylpolyene, sactipeptide, nonribosomal peptide synthetase (NRPS), bacteriocin, terpene, betalactone, resorcinol, and lantipeptide (Figure 1A). We subsequently explored the variations in the microbial BGCs’ composition from different intestinal segments. The specific and shared metabolite genes are shown in a Venn diagram (Figure 1B). In detail, the comparative analyses revealed that four types were unique to the ileal microbiome, including resorcinol, ectoine, RaS-RiPP, and hserlactone. However, polyketide synthase (PKS), ladderane, and LAP only were observed in the colonic microbiome, but not in the ileal microbiome. The secondary metabolite genes from the ileum or colon shared 12 BGC types. Arylpolyene (15.48%, 34.92%), sactipeptide (22.62%, 14.92%), NRPS (16.07%, 16.27%), and bacteriocin (20.24%, 10.85%) were both present in high proportions in ileal and colonic contents, respectively.

**Figure 2:**
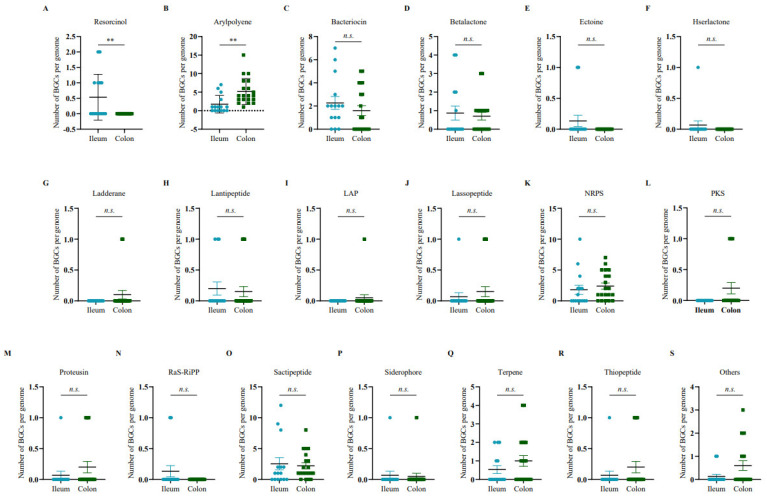
The number of different BGCs from ileum and colon, including (**A**) resorcinol, (**B**) arylpolyene, (**C**) bacteriocin, (**D**) betalactone, (**E**) ectoine, (**F**) hserlactone, (**G**) ladderane, (**H**) lantipeptide, (**I**) LAP, (**J**) lassopeptide, (**K**) NRPS, (**L**) PKS, (**M**) proteusin, (**N**) RaS-RiPP, (**O**) sactipeptide, (**P**) siderophore, (**Q**) terpene, (**R**) thiopeptide, and (**S**) others. Statistical analyses were performed using two-tailed unpaired *t*-tests. ** *p* < 0.01, *n.s.*, nonsignificant differences.

Furthermore, the gene abundance of resorcinol was markedly increased in the ileal microbiome (Figure 2A), and the gene clusters of arylpolyene showed enrichment in the colonic microbiome (Figure 2B), while there was no significant difference in other secondary metabolite genes between the ileum and colon (Figure 2C–S).

**Figure 3:**
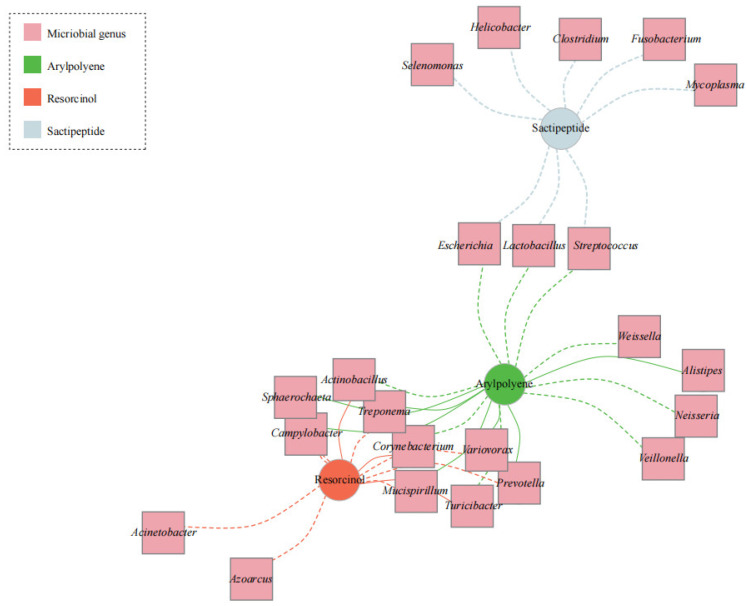
A network integration of intestinal microbiome and secondary metabolite genes. A positive correlation between nodes is indicated by the solid line, and negative correlation by the dashed line. The circles represent metabolites, and the pink squares represent genera with significant differences by Spearman’s rank correlation coefficient more than 0.5 and probability value less than 0.05.

We also undertook a network analysis based on the predicted secondary metabolites and microbial genera from the metagenomic datasets in all samples to look for broader associations. Three clusters were defined between the BGCs and microbiomes (Figure 3). Sactipeptide showed a negative association with eight bacterial genera. Based on the association mapping, six bacterial genera (*Variovorax*, *Campylobacter*, *Mucispirillum*, *Prevotella*, *Sphaerochaeta,* and *Treponema*) were enriched in the arylpolyene cluster and depleted in the resorcinol cluster. *Actinobacillus*, *Corynebacterium,* and *Turicibacter* were positively associated with resorcinol but negatively correlated with arylpolyene.

## 4. Discussion

In the veterinary field, the misuse of antibiotics is a serious health challenge and imposes a strong selective pressure on bacteria [8,30,31]. With the rapid progress of sequencing techniques, more and more high-quality metagenomic sequencing of genetic resources in complex microbial communities has been completed [32], which is analogous to genome library construction and screening [33]. Through the mining of gene sequencing information in the past decades, a large number of reagents composed of enzymes [33], small peptides [34], and secondary metabolites [35,36,37] have been discovered and applied in industry, medicine, and agriculture. Here, we revealed the natural products from metagenomic sequencing in the pig gut.

Natural microbial products are abundant sources of therapeutic agents for animal and human health [38]. They are constructed and tailored by BGCs in various microorganisms, especially actinomycetes, cyanobacteria, and myxobacteria [39]. These BGCs are generally organized into multiple operon modules within genomes, which work together as assembly lines to tailor metabolic products [40]. In inter- and intraspecies interactions, natural products participate in biofilm inhibition [41], stress protection [42], and the killing of competitors [37]. To address the threat from multiple-resistant bacteria, molecules with growth-inhibitory activities have aroused the specific interest of researchers and may serve as precursors to the lead compounds of new antibiotics. A bacteriocin LSB1 purified from *Lactobacillus plantarum* CGMCC 1.12934 exhibited an extensive antimicrobial spectrum against both Gram-positive and Gram-negative bacteria, and significantly impaired the biofilm formation ability of *Staphylococcus argenteus* [43]. In genome mining, nonribosomal peptide synthetases, polyketide synthases, and the ribosomally synthesized and post-translationally modified peptides are the most well-characterized BGCs [44,45,46].

In our study, there were diverse secondary metabolite genes in the porcine intestine (Figure 1). The abundance of arylpolyene was increased in the colonic microbiome (Figure 2A); conversely, the content of resorcinol in the ileum microbiome significantly increased compared with that in the colon microbiome (Figure 2B). It was reported that resorcinol has antimicrobial, anti-inflammatory, and antioxidant effects [47,48,49]. Using LC-MS/MS and LC/MS^n^ -IT-TOF techniques, Adili Keranmu et al. reported that gut microbiota could metabolize pharmacological flavonoids into resorcinol (1,3-dihydroxybenzene) [50]. Resorcinol suppressed aryl hydrocarbon receptor activity, as evidenced by the inhibition of B[a]P-induced xenobiotic response element reporter activation and cytochrome P450 1A1 expression [49]. In this study, a network diagram of correlation was drawn to identify representative microorganisms for different BGCs (Figure 3). Network analysis revealed that the bacterial populations correlated with resorcinol, including eight bacterial genera (*Azoarcus*, *Acinetobacter*, *Campylobacter*, *Mucispirillum*, *Prevotella*, *Sphaerochaeta*, *Treponema,* and *Variovorax*) with a negative association and three bacterial genera with a positive association. The denitrifying bacterium *Azoarcus* has received considerable attention with unusual reactions to degrade aromatics. In anaerobic conditions, resorcinol functions as a sole carbon and energy source for bacterial growth [51], which might explain the resorcinol reduction in the intestinal samples with an increased abundance of bacterium *Azoarcus*. For arylpolyene, there were positive associations with the abundances of seven bacterial genera. For example, *Variovorax* was enriched in samples with a high level of arylpolyene, which is consistent with the findings in a previous study. Tim et al. found the production of arylpolyene/dialkylresorcinol hybrid pigment from *Variovorax paradoxus* B4 and elucidated the polyene structure [52].

Synthetic gene clusters of arylpolyene are widely found in bacteria, including human commensals, animal pathogens, and plant pathogens and symbionts [52]. Arylpolyene is a lipid with an aryl head group and a polyene carboxylic acid tail [53]. The main scaffold differences among different bacterial genera lie in the chain length of polyenes and the modification methods of head genes, including hydroxylation, methylation, and halogenation [54]. The conservative chemical structure indicates that these compounds might play an important role in microbial physiological activities [54]. The compound is structurally similar to carotenoids and has a similar function to carotenoids to protect bacteria from reactive oxygen species [52]. For example, membrane-bound arylpolyene was reported to reduce the concentration of free radicals in ecological niches and protect cells from oxidative stress damage related to lipids, proteins, and nucleic acids [54]. Furthermore, arylpolyene was found to promote the biofilm formation in pathogens [54].

Our results revealed the existence of a large number of BGCs in the pig gastrointestinal tract and the heterogeneity in the distribution of these BGCs. Resorcinol has an antimicrobial property, and arylpolyene has an antioxidant property. Yet, there were some limitations in this study. The roles of arylpolyene and resorcinol remain to be clarified and verified, and their expression levels and activities must be further examined through metatranscriptomics and metabolomics. Although genome mining could quickly and easily predict BGCs from metagenomics data, gene expression is a better approach for functional activity research [55,56]. Metatranscriptomics is complementary to metagenomics sequencing, which can elucidate the characteristics of microbial communities and provide some information for the accurate annotations of genes and their regulation in their community [57]. So far, few secondary metabolites from gut microbes have been well characterized. First, the majority of bacteria from nature are currently unculturable with traditional laboratory techniques [58]. Second, the expression of bioactive secondary metabolites may remain silent or at a relatively low level [59]. Third, how to separate and access these low-expressed secondary metabolites from a large number of complex metabolites in the animal gut has puzzled researchers for quite a long time [60]. In the future, our findings need further analysis and validation at the transcriptional and metabolic levels.

## 5. Conclusions

In conclusion, our findings showed that there are diversified BGCs in the pig gastrointestinal tract. In addition, the heterogeneous distribution of BGCs revealed that gene clusters of resorcinol and arylpolyene were enriched in the ileal and colonic microbiomes, respectively. These findings lay a foundation to exploit veterinary antibiotic alternatives from highly complex and diverse gut microbiomes. Nevertheless, the roles of arylpolyene and resorcinol remain to be clarified by metatranscriptomics and metabolomics.

## Figures and Tables

**Table 1:** Ingredient composition and chemical analysis of the weaning piglet diet.

Ingredients	Content, g/kg
Corn	600
Soybean meal	230
Wheat bran	50
Fish meal	20
Whey	50
Soybean oil	10
Premix ^1^	40
Chemical compositions	
Digestible energy ^2^, MJ/kg	13.80
Metabolizable energy ^2^, MJ/kg	12.77
Crude protein ^3^, %	18.50
Lysine ^3^, %	1.18
Methionine ^3^, %	0.32
Calcium ^3^, %	0.76
Total phosphorus ^3^, %	0.56

^1^ Provided per kilogram of complete diet: vitamin A, 10,000 IU; vitamin D3, 2700 IU; vitamin E, 28 mg; vitamin K3, 2 mg/kg; vitamin B1, 2 mg/kg; vitamin B2, 5 mg; vitamin B6, 2.5 mg; vitamin B12, 0.04 mg; niacin, 30 mg; pantothenic acid, 12 mg; folic acid, 1 mg; biotin, 0.5 mg; Fe, 130 mg; Zn, 130 mg; Cu, 160 mg; Mn, 20 mg; I, 0. 5 mg; Se, 0.3 mg. ^2^ Calculated nutrient levels. ^3^ Measured nutrient levels.

## Data Availability

The whole-genome sequence data reported in this paper were deposited in the Genome Warehouse in the National Genomics Data Center, Beijing Institute of Genomics, Chinese Academy of Sciences/China National Center for Bioinformation (GWH: GWHBOXZ00000000-GWHBOZH00000000), and are publicly accessible at https://ngdc.cncb.ac.cn/gwh, accessed on 26 October 2022.
